# *MXCuBE2*: the dawn of *MXCuBE* Collaboration

**DOI:** 10.1107/S1600577519001267

**Published:** 2019-02-22

**Authors:** Marcus Oscarsson, Antonia Beteva, David Flot, Elspeth Gordon, Matias Guijarro, Gordon Leonard, Sean McSweeney, Stephanie Monaco, Christoph Mueller-Dieckmann, Max Nanao, Didier Nurizzo, Alexander N. Popov, David von Stetten, Olof Svensson, Vicente Rey-Bakaikoa, Idrissou Chado, Leonard M. G. Chavas, Laurent Gadea, Patrick Gourhant, Tatiana Isabet, Pierre Legrand, Martin Savko, Serena Sirigu, William Shepard, Andrew Thompson, Uwe Mueller, Jie Nan, Mikel Eguiraun, Fredrick Bolmsten, Alberto Nardella, Antonio Milàn-Otero, Marjolein Thunnissen, Michael Hellmig, Alexandra Kastner, Lukas Schmuckermaier, Martin Gerlach, Christian Feiler, Manfred S. Weiss, Matthew W. Bowler, Alexandre Gobbo, Gergely Papp, Jeremy Sinoir, Andrew A. McCarthy, Ivars Karpics, Marina Nikolova, Gleb Bourenkov, Thomas Schneider, Jordi Andreu, Guifré Cuní, Judith Juanhuix, Roeland Boer, Rasmus Fogh, Peter Keller, Claus Flensburg, Wlodek Paciorek, Clemens Vonrhein, Gerard Bricogne, Daniele de Sanctis

**Affiliations:** a ESRF - The European Synchrotron, 71 Avenue des Martyrs, 38000 Grenoble, France; b TXO Pedro de Alejandria 2-2C Pamplona, Spain; c Synchrotron SOLEIL, 91192 Gif-sur-Yvette Cedex, France; d MAX IV Laboratory, Lund University, SE-221 00 Lund, Sweden; e Helmholtz-Zentrum Berlin für Materialien und Energie, Macromolecular Crystallography, Albert-Einstein-Straße 15, D-12489 Berlin, Germany; f European Molecular Biology Laboratory, Grenoble Outstation, 71 Avenue des Martyrs, 38042 Grenoble, France; g Hamburg Unit c/o DESY, European Molecular Biology Laboratory (EMBL), Notkestrasse 85, 22603 Hamburg, Germany; h CELLS-ALBA Synchrotron Light Source, 08290 Cerdanyola del Vallès, Spain; i Global Phasing Ltd, Sheraton House, Castle Park, Cambridge CB3 0AK, UK

**Keywords:** macromolecular crystallography, synchrotron beamline control software, software collaboration, graphical user interface, *MXCuBE*

## Abstract

*MXCuBE2* is the second-generation evolution of the *MXCuBE* beamline control software, initially developed and used at ESRF – the European Synchrotron. *MXCuBE2* extends, in an intuitive graphical user interface (GUI), the functionalities and data collection methods available to users while keeping all previously available features and allowing for the straightforward incorporation of ongoing and future developments. *MXCuBE2* introduces an extended abstraction layer that allows easy interfacing of any kind of macromolecular crystallography (MX) hardware component, whether this is a diffractometer, sample changer, detector or optical element. *MXCuBE2* also works in strong synergy with the *ISPyB* Laboratory Information Management System, accessing the list of samples available for a particular experimental session and associating, either from instructions contained in *ISPyB* or from user input via the *MXCuBE2* GUI, different data collection types to them. The development of *MXCuBE2* forms the core of a fruitful collaboration which brings together several European synchrotrons and a software development factory and, as such, defines a new paradigm for the development of beamline control platforms for the European MX user community.

## Abstract 2

The collaboration for the development of *MXCuBE2* control software for Macromolecular Crystallography beamlines is described

## 1. Introduction   

The past 15 years or so have seen a dramatic increase in the productivity of synchrotron macromolecular crystallography (MX) beamlines, both in terms of the number of crystals analysed and the number of new crystal structures deposited in the Protein Data Bank (PDB) (Berman *et al.*, 2000 ▸). This evolution has, to a large extent, been driven by improvements in beamline hardware and software. In particular, the smaller X-ray beams provided by third-generation synchrotron sources have opened new avenues in MX, making it possible to collect diffraction data, using increasingly advanced experimental protocols, from smaller crystals of increasingly intricate and important structural biology targets. However, in order that experimenters can take best advantage of the increasingly complex data collection options available, a robust, user-friendly and adaptable beamline control graphical user interface (GUI) is essential.

In 2006, the ESRF and the Medical Research Council UK (MRC-UK) started to develop the *MXCuBE* beamline control GUI (Gabadinho *et al.*, 2010 ▸) with the aim of providing the users of its various MX facilities with an almost identical user-control environment regardless of any differences in beamline hardware. *MXCuBE* was initially deployed both at the ESRF MX beamlines and at the Collaborative Research Group (CRG) beamline BM14. This was quickly followed by installations on the MAX-lab beamline I911-3 and the Helmholtz-Zentrum Berlin (HZB, BESSY II) beamline BL14.1 (Mueller *et al.*, 2012 ▸). However, installation at sites other than the ESRF was not always straightforward due to differences in the low-level beamline server and control software installed. Discussions concerning the development of the second-generation *MXCuBE2*, designed to be independent of both beamline hardware and lower-level server and control software, were thus instigated and the *MXCuBE* Collaboration was formed (§2). The *MXCuBE2* GUI resulting from the collaboration was also simplified, permitting a closer interaction with the sample under study while, at the same time, including a growing number of new MX experimental methodologies. In particular and by design, *MXCuBE2* is able to interact with external ‘experiment descriptors’ (Brockhauser *et al.*, 2012 ▸) which can iteratively define and adjust data collection parameters according to results obtained by connected analyses. Although there is no reciprocal dependency, user experience is greatly enhanced by an increased synergy between *MXCuBE2* and the *ISPyB* Laboratory Information Management System (LIMS) (Delagenière *et al.*, 2011 ▸). To better interact with *MXCuBE2*, *ISPyB* has been expanded to include fuller sample descriptors and ‘diffraction plans’ and now stores all the details of all the experiments (including the results of on-line data analyses) performed on a given sample.

The major aim of the *MXCuBE* Collaboration is the pooling of resources to provide a standardized and user-friendly beamline control GUI at the synchrotron sites involved and to provide a platform for the rapid implementation, at all sites where these are desired, of new functionality and experimental protocols. In this context, we present here details of the collaboration, of *MXCuBE2* itself and of the contribution of the different partners making up the consortium.

## 2. The *MXCuBE* Collaboration   

Experience gained at ESRF, first with *ProDC* (Arzt *et al.*, 2005 ▸), then with initial versions of *MXCuBE* (Gabadinho *et al.*, 2010 ▸), showed that the development, maintenance and continuous upgrade of GUIs for the control of experiments at synchrotron-based MX beamlines is an extremely labour-intensive endeavour requiring significant human resources from beamline scientists and software engineers. This is particularly the case when the beamline control GUI is linked to synchrotron-based LIMS such as *PXWeb* (Arzt *et al.*, 2005 ▸) or *ISPyB* (Delagenière *et al.*, 2011 ▸).

In Grenoble, the *ProDC*/*PXWeb* combination for synchrotron beamline control/experiment-tracking was developed by the ESRF–EMBL Joint Structural Biology Group (JSBG) while initial versions of the *MXCuBE*/*ISPyB* combination were developed in collaboration with the MRC-UK operated BM14 CRG. The JSBG–BM14 link-up had two aims: in tandem with funding from the BIOXHIT [Biocrystallography (X) on a Highly Integrated Technology Platform for European Structural Genomics] project, the collaboration helped ensure the availability of sufficient human resources to develop, maintain and upgrade *MXCuBE*/*ISPyB* and, at the same time, provided a standard beamline control GUI for experiments at eight ESRF-based MX beamlines. The MRC-UK BM14 CRG ceased operating in 2011. In addition to the ESRF, *MXCuBE* was also installed on MX beamlines at the MAX-II, HZB (BESSY II), EMBL-Hamburg and SOLEIL synchrotron sites who, along with the protein crystallography software company Global Phasing Ltd, continued to provide ideas and human resources for its development. This informal collaboration was officialized by the signing in 2012 of a Memorandum of Understanding (MoU), the aims of which were to define the organization of a collaboration, to oversee the installation, support, documentation and further development of *MXCuBE* at several European Synchrotron sites, to present a similar and familiar interface for MX experiments at all of the synchrotron sites involved, and to share the human resources costs of the development, support and maintenance of the software. The collaboration has continued to expand and the partners now comprise ESRF, EMBL, Synchrotron SOLEIL, HZB (BESSY II), ALBA, DESY, MAX IV Laboratory and Global Phasing Ltd, while Elettra and LNLS are in the process of joining the collaboration.

Management of the collaboration is handled by three committees: the *MXCuBE* Steering Committee (STC), the *MXCuBE* Scientific Committee (SC) and the *MXCuBE* Developers Committee (DC). As a minimum condition for joining the collaboration each partner is required to assign a software engineer who should contribute at least 10% of working time to developments carried on within the collaboration. The role of the STC is to define the long-term strategy of the collaboration ensuring, in particular, adequate manpower and financial resources for the smooth functioning of the collaboration. The SC defines the scientific requirements and development priorities for *MXCuBE* while members of the DC are charged with implementing software developments requested by the SC. The DC also ensures that at any given time there is only a single official *MXCuBE* version, available through a publicly accessible website for download and collaboration on source code (https://github.com/mxcube; https://www.mxcube.org). The STC usually convenes only at biannual *MXCuBE* Collaboration meetings, organized by each of the partners in a round-robin fashion, at which progress is presented and strategic issues discussed. Meetings of the SC and the DC are held more frequently with, outside of the *MXCuBE* Collaboration meetings, sessions organized on an *ad hoc* basis as and when necessary. New members of the collaboration may join at any time. All that is required is the agreement of the STC and the subsequent signing by the new partner of the *MXCuBE* MoU.

The setting up of the *MXCuBE* Collaboration has provided, to the benefit of the European structural biology community, a stable, productive environment for the development of *MXCuBE*. Such has been the success of the collaboration that its MoU-based membership and management structure have now been adopted by the *ISPyB* Collaboration (https://github.com/ispyb) recently set up to oversee the joint development of the *ISPyB* LIMS.

## 3. The design of *MXCuBE2*   

**Figure 1:**
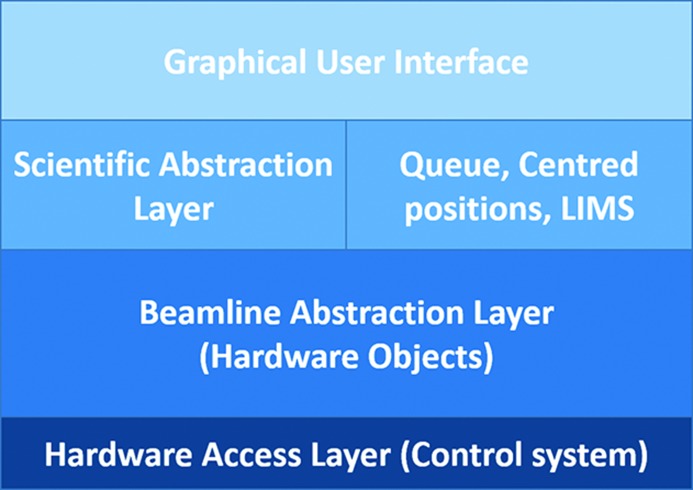
*MXCuBE2* architecture. The GUI gives access to the scientific abstraction layer (data collection methods or external ‘experiment descriptors’) and manages the queue execution, centred positions and communicates with the LIMS. A beamline abstraction layer constituted by the hardware objects ensures the control of the hardware access layer independently from the control system used.

*MXCuBE2* comprises two main components: a GUI and a data acquisition control layer. The *MXCuBE2* GUI, similarly to its predecessor, is built using the Bliss framework (Guijarro *et al.*, 2004 ▸; http://github.com/mxcube/BlissFramework), a tool developed at the ESRF for building graphical interfaces for beamline instrumentation control and based on Python and the Qt3 toolkit. Porting to more recent versions of Qt (versions 4 and 5, respectively) has been carried out within the *MXCuBE* Collaboration and has allowed the extension of the lifetime of the *MXCuBE2* GUI beyond the official support provided by the Qt3 packages in different Linux distributions. The GUI is built by combining different graphical widgets named bricks. Bricks are individual graphical objects integrated in the GUI. They are connected to hardware objects that directly control and display information such as the position and the status of the beamline equipment (Fig. 1 ▸).

### 3.1. Beamline abstraction layer – hardware objects   

Hardware objects are Python classes directly associated with a configuration eXtensible Markup Language (XML) file which make the link between beamline physical devices and the user interface graphical components. Hardware objects are based on abstract classes and are accessed through a common application programming interface (API) from *MXCuBE2*. This allows the straightforward adaptation of *MXCuBE2* to the different hardware and beamline specificities of different synchrotron sites.

Hardware object XML configuration files can be made available either by a Hardware Repository Server or just read from a directory. The added benefit of the Hardware Repository Server is that it allows shared read and write access to hardware objects. Indeed, when the server modifies XML files it can send notifications to listening instances of *MXCuBE2*, which can, in turn, reload hardware objects accordingly.

Hardware objects can contain other hardware objects, thus enabling the more complete representation of complex hardware and equipment in a generic manner. The life cycle of Python hardware objects is as follows: at start-up, *MXCuBE2* gets the list of hardware objects necessary to create the user interface components. The set of Python hardware objects is then instantiated from the information contained in hardware repository files. Finally, hardware objects are passed to the corresponding user interface components. Python hardware objects are singletons within a running *MXCuBE2* instance, meaning that the objects are shared among all user interface components. Only one unique instance of each hardware object lives in a running *MXCuBE2* application. When an *MXCuBE2* session is terminated, the hardware objects are ended and destroyed.

Multiple *MXCuBE2* applications (such as in remote access, §4.5) create their own set of hardware objects. The current hardware objects model in *MXCuBE2* assumes concurrent access to beamline devices from different processes and is managed at the beamline control system level.

### 3.2. The *MXCuBE2* queue   

**Figure 2:**
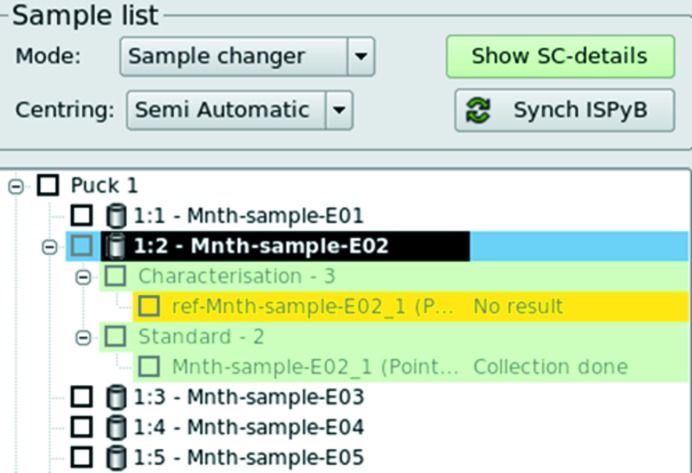
The GUI representation of the *MXCuBE2* queue. Each sample present in the sample changer dewar is represented as a node, while child nodes group data collection on that sample.

The *MXCuBE2* queue (Fig. 2 ▸) holds the sequence of operations to be executed by a user carrying out an experiment at an MX beamline. One of the core ideas of *MXCuBE2* is to represent all data acquisition sequences, including sample loading and unloading operations, as tasks in a tree structure. At the first level of the *MXCuBE* queue, tree nodes represent samples. At the second level, data collection group nodes contain logically grouped tasks; for example, inverse beam data collection tasks, or other interleaved data collection procedures (*e.g.* an absorption-edge scan followed by data collection at peak and inflection points *etc*.). Tasks are the leaf children nodes of the *MXCuBE2* queue. In this way, different data collections on a single or multiple samples can be chained in a time-efficient manner. More complex data collection protocols can also be prepared and executed with minimal user intervention. The construction of a queue puts the focus on data collection rather than sample loading and unloading procedures, which is an intrinsic step for data collection on a sample.

**Figure 3:**
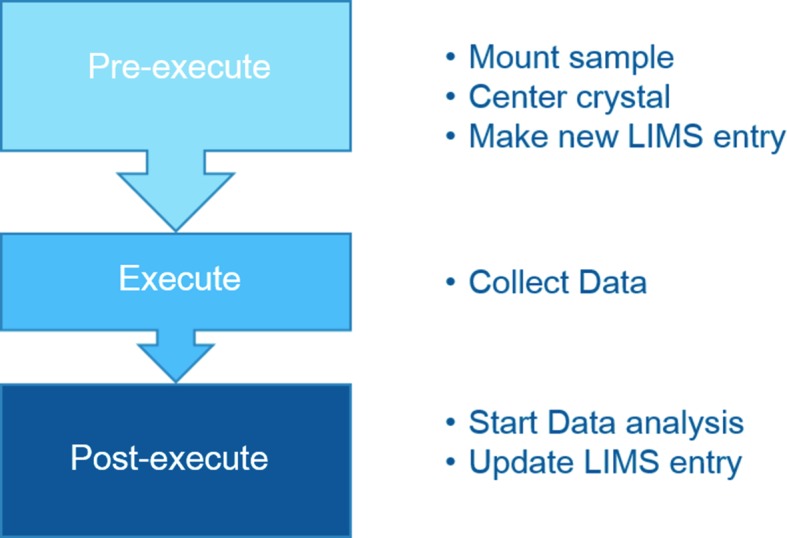
The three phases of the execution of a data collection on a selected sample. Unless already mounted, the pre-execute controls the sample changer. Once the sample is mounted, a centred position is selected, automatically or by the user, and the experiment details are then uploaded to the LIMS. After the data collection, the post-execute triggers the data analysis and updates the LIMS entry with the data collection status.

The queue is split into three main components: manager, queue entries and model objects. This allows isolating queue execution (QueueManager object), task logic (QueueEntry objects) and task description (QueueModel objects) from each other, ensuring a clean separation of the involvement of the different objects and securing a straightforward serialization of the queue either to disk or via the network (§3.3). In fact, QueueEntry objects are composed of both QueueModel objects and hardware objects. The latter are live Python objects with connections to hardware devices (§3.1) which cannot be self-described with basic data types. Model objects specify the kind of task to be executed and its associated data, whereas queue entries contain model objects (tasks) and the corresponding hardware objects to execute the task described in the model. QueueEntry objects define three main methods for implementing the execution logic for each task: ‘pre-execute’, ‘execute’ and ‘post-execute’ (Fig. 3 ▸). For example, a sample queue entry will operate the sample changer device to load the sample in the ‘pre-execute’ method, and will unload the sample in the ‘post-execute’ method. In addition to queue entry objects, the QueueManager is directly associated with the queue user interface component. Its role is to go through the tree of queue entries, and to execute them one by one as configured by the user, using queue controls from the user interface or from a third-party tool such as an ‘experiment descriptor’.

### 3.3. XML-RPC server   

*MXCuBE2* embeds an XML-RPC server allowing access to high-level *MXCuBE2* queue methods by external, third party software. This concept is another key feature of *MXCuBE2*, permitting the creation of new data collection protocols that would be tedious to set up manually. As a result of this organization, an external ‘experiment descriptor’ tool represented as an expert system can be very beneficial. The Remote Procedure Call service provided by the embedded XML-RPC server exposes the queue API to access core components of *MXCuBE2*, just like a single user would do using the queue control from the user interface. XML-RPC was chosen for its simplicity and its availability in many languages (C, C++, Java, Python and others). Indeed, an XML-RPC client for *MXCuBE2* could be written in another language than that of *MXCuBE2* itself. The data exchange between XML-RPC clients and the embedded XML-RPC server is performed using remote procedure calls, as specified by the XML-RPC standard and serialized *MXCuBE2* QueueModel objects. The model objects are serialized in JSON (JavaScript Object Notation) format. The exposed API allows the creation and manipulation of queue entries and the assignment of these entries’ model objects as tasks for execution. The queue manager object API is also exposed in order to be able to perform start and stop actions, and monitor queue execution.

## 4. GUI description   

**Figure 4:**
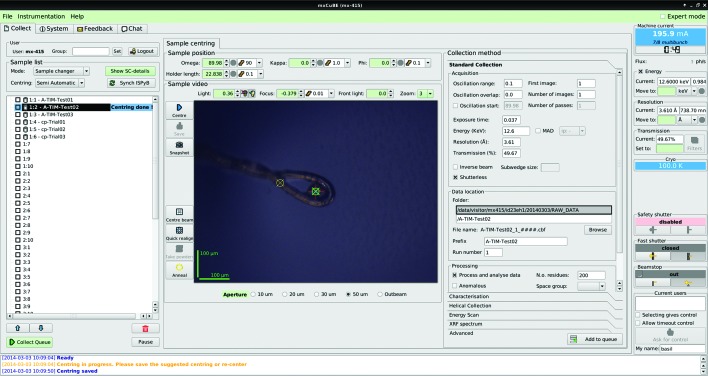
Overview of *MXCuBE2* (using Qt3 framework). As described in the main text, four main areas constitute the GUI. The currently mounted sample is highlighted (while centred positions are tracked on screen) in green and yellow encircled crosses for active and inactive positions, respectively. The collection methods are organized in vertical tabs, each containing the most relevant data collection parameters.

The *MXCuBE2* GUI presents all of the most fundamental operations required during an experiment in a single window, allowing users to mount samples and perform various kinds of data collection in an intuitive way. The GUI is split into four main areas (Fig. 4 ▸). The left column contains a login area (requiring site-specific credentials) and the sample list where the contents of the sample changer are permanently displayed and accessible. Users must login to the GUI in order to have access to, and to obtain control of, the sample changer and the sample list content. After logging in, a group identifier facilitates backing up and retrieval of the data by storing all data in a common folder named after the indicated group name. This option is particularly useful in the case of different groups sharing their beam time and proposal number, as often happens at the ESRF when scheduling users from the Block Allocation Group (BAG).

The central part of the GUI accommodates widgets for controlling the goniometer motors and for the sample visualization microscope view. The collection methods area then presents six main groups: ‘Standard data collection’, ‘Characterization’, ‘Helical’, ‘Energy scan’, ‘XRF’ and ‘Advanced’ tabs can be accessed depending on the requirements of an experiment. The right column of the GUI groups a set of information widgets. The top part of this panel displays the information from the storage ring (such as stored ring current, filling mode, time to next injection), the photon flux at the sample position and the temperature of the cryo-system. Underneath this area, experimental parameters such as incident X-ray energy (or wavelength), resolution (or sample-to-detector distance) and transmission are displayed and their values can be changed in their respective motor or actuator bricks. Finally, widgets allowing the control and monitoring of beam shutters, the control and monitoring of the beam-stop position, and tools for remote access are displayed. A concise log at the bottom of the window displays information messages and beamline events. Three additional (and optional) tabs help users to access functionalities only occasionally used, *i.e.* the ‘System log’ (containing a more verbose logging of the events), a ‘Feedback’ widget for sending emails to the local staff and a ‘Chat’ function allowing live communication with remotely logged users.

### 4.1. Sample changer and sample list   

**Figure 5:**
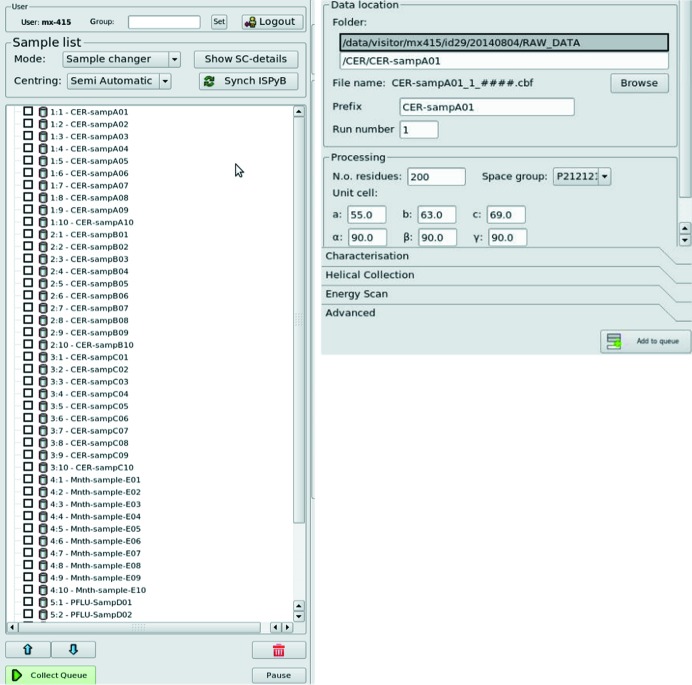
(Left) The *MXCuBE2* sample list after synchronization with *ISPyB* (via the ‘Synch *ISPyB*’ button) represents each sample by its name. (Right) Data are automatically saved in a unique folder and file name. When a space group and unit cell is specified in *ISPyB*, this information is used and transmitted to the auto-processing pipelines.

The sample list displays all the sample positions available within the sample changer installed on a given beamline. Once the sample changer has been loaded and the positions of the pucks noted then, provided the relevant information has been pre-recorded and stored in *ISPyB*, clicking the ‘Synchronize *ISPyB*’ button will fill the sample list with a complete description of the contents (on a crystal level) of the sample changer (Fig. 5 ▸, left). This information is used to automatically create a folder and subfolder structure (/local/path/to/images/"protein"/"protein"-"sample"/) and diffraction image files with appropriate file names (Fig. 5 ▸, right). The sample currently mounted is highlighted in the sample list for immediate visualization/retrieval (Figs. 2 ▸ and 4 ▸). Data collection methods for each crystal can be added and displayed as nodes of a tree directly at the selected sample location in the list (§3.2). When an operation is performed, the background is then coloured either in green (successful), yellow (completed, but without results) or red (failure in the data collection process). Samples can be mounted directly from the sample list (by right clicking on the targeted sample). The execution of chain-loading procedures (*i.e.* tacit unloading of the current sample by directly loading the following one) is possible and recommended for sample changers requiring de-freezing cycles between explicit operations. If multiple samples are selected and data collection methods added to them, the queue will be executed in pipeline mode and the next sample automatically mounted when the list of tasks for the previous sample is successfully completed. The queue can be stopped or paused at any time by pressing a corresponding button if any modification is required. More advanced functionalities of the sample changer (motor homing, gripper changing, troubleshooting) are accessible by clicking on a dedicated button (‘Show SC’) that opens a new tab.

### 4.2. Sample viewing, saving position and mesh tool   

**Figure 6:**
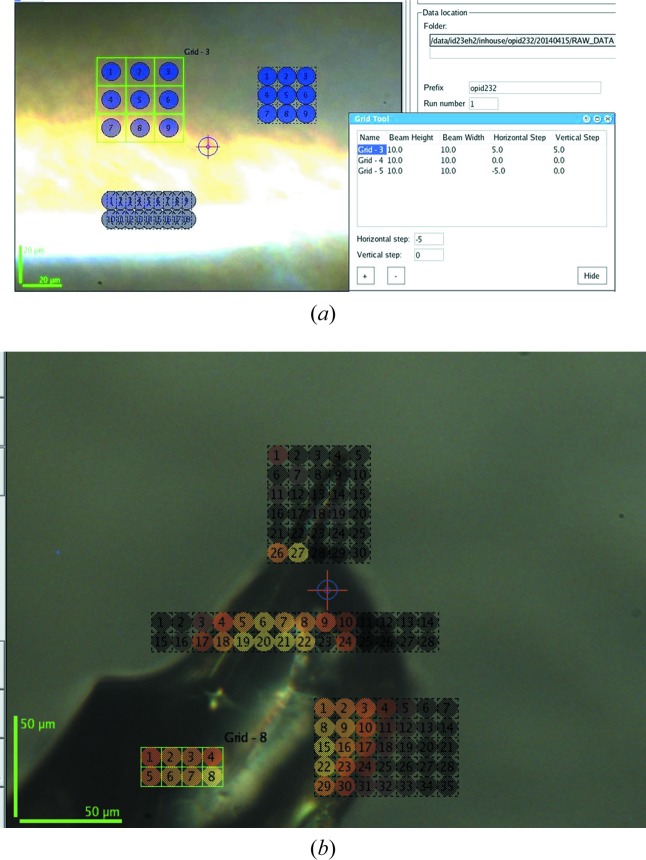
(*a*) Examples of the different mesh tools, with vertical and horizontal spacing defined in the Grid tool dialogue box. (*b*) After data analysis, grid points are coloured according to the mesh analysis score.

The sample view displays a video of the sample retrieved through the camera of the sample microscope viewer. The video is always visible, centralizing the user interaction on the mounted sample. To facilitate the use of small X-ray beams to collect (partial) data sets at more than one position in a crystal, *MXCuBE2* introduces the possibility to save multiple positions directly on screen and to relate each position in the laboratory and camera frame to the corresponding alignment and positions of the centring motors. Each centred position is tracked and displayed on the screen by a yellow encircled cross, while selected and active saved positions are shown in bold green. It is possible to activate positions sequentially and to add in the queue the same, or different, data collection protocols to different activated positions. The location of one or more small crystals on the same support, or the determination of the best diffracting volume of a large crystal, required the development of dedicated tools that allow rastering through a two-dimensional mesh drawn across the sample support. Originally a very time-consuming procedure, the advent of photon-counting detectors coupled to the synchronized movement of multiple motors (trajectories) led to the implementation of linear scans across the grid by horizontal lines (vertical for upward or downward spindles) while the detector is continuously acquiring data. Eventually, this further evolved in the implementation of a serpentine-like scan across the grid with the detector being triggered in correspondence of each mesh point. The grid drawing tool available in *MXCuBE2* uses the current beam size as the default unit for calculating the size and number of grid points in a mesh scan, while it is also possible to specify spacing or overlaps between the measured positions (Fig. 6 ▸).

### 4.3. Data collection methods   

The sample list displays data collections that have been performed and those that are currently queued. Data collection operations are assigned to saved positions on a given crystal. Once an operation or a series of operations are assigned to a saved position, if these are still queued (*i.e.* not yet executed) it is possible to interactively delete or modify these by clicking on the data collection entry, then clicking on the ‘Remove’ or ‘Details’ sub-tab. In the latter, the parameters associated with a data collection can be edited. Some operations [*i.e.* ‘Characterization’ (§4.3.2)] generate a new entry in the data collection tree. In such cases, the user has the choice of automatically launching the new entry or reviewing and/or modifying the experimental parameters generated (again via the ‘Details’ sub-tab) before the data collection is launched.

#### 4.3.1. Standard data collection   

‘Standard’ data collection specifies a ‘normal’ oscillation data collection (*i.e.* a contiguous total rotation range) operation performed at a single centred point on the crystal of interest. In addition to usual parameters (omega start, exposure time, oscillation, number of frames), it is possible to specify the resolution and the energy at which the diffraction measurements are to be performed. In case the energies for the peak (*f*′′ max), inflection point (*f*′ min) and remote were determined from an absorption-edge scan performed on the selected sample, it is possible to select them directly from a MAD dropdown menu within the same widget. Optionally, the space group and unit cell of the crystal can be indicated for use in downstream automatic data-processing pipelines [*i.e.* *GrenADES* (Monaco *et al.*, 2013 ▸); *XDSAPP* (Sparta *et al.*, 2016 ▸); *autoPROC* (Vonrhein *et al.*, 2011 ▸), *etc.*].

#### 4.3.2. Characterization   

Characterization of crystals has the scope of determining an optimal standard data collection strategy that fulfils certain requirements, most notably the taking into account of radiation damage to a sample during a data collection, defined by the user. The *MXCuBE2* characterization pipeline is embedded into the *EDNA* framework (Incardona *et al.*, 2009 ▸) and links software for the indexing and/or integration of diffraction images (*MOSFLM*, *LABELIT*, *XDS*) (Leslie & Powell, 2007 ▸; Sauter & Poon, 2010 ▸; Kabsch, 2010 ▸), absorbed dose calculation/prediction (*RADDOSE*) (Paithankar & Garman, 2010 ▸) and data collection strategy calculation (*BEST*) (Bourenkov & Popov, 2010 ▸). The program *XOalign* (Legrand, unpublished; https://github.com/legrandp/xdsme/tree/master/XOalign) calculates possible crystal reorientation using kappa goniometry that can be exploited further for a new strategy calculation.

The *EDNA* characterization module relies on various inputs, such as crystal and beam size, and the photon flux impinging on the sample. In most cases this is either stored as metadata in *ISPyB* and retrieved for analysis or provided directly by *MXCuBE2*. A successful characterization produces a ‘diffraction plan’, automatically added to the data collection queue. If required, the parameters for the diffraction plan data collection can be edited by the users via the ‘Details’ sub-tab before execution (§4.3).

#### 4.3.3. Helical data collection/characterization   

Helical (also known as vector) data collection is performed by continuously rotating a crystal while, at the same time, translating it along a defined line or vector (Flot *et al.*, 2010 ▸). Thus, an undamaged part of the crystal is continuously exposed to the X-ray beam, allowing for the use of higher absorbed dose limits than would be the case for a standard data collection. In *MXCuBE2*, the line along which the crystal is translated is defined by two previous centred and saved positions on the crystal. Once these are chosen, the user then defines data collection parameters as for a standard data collection. At the ESRF, to help ensure that the absorbed dose is optimized as a function of beam size and vector length, users have the option, found in the ‘Advanced’ data collection tab (§4.3.5), of carrying out a ‘Helical Characterization’. Upon addition of this option to the data collection queue and its subsequent execution, a data collection ‘wizard’ guides the user through the setting up of the experiment, including the choice of centred positions to be used to define the vector. Once these are defined and saved, *MXCuBE2* automatically carries out a characterization at the mid-point between the two positions and calculates an optimal diffraction plan (§4.3.2).

#### 4.3.4. Absorption-edge scan and X-ray fluorescence spectra   

**Figure 7:**
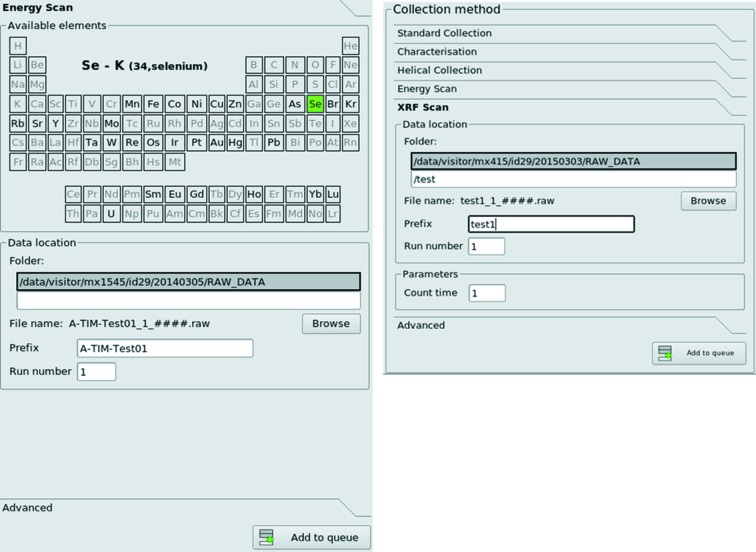
Energy scan and X-ray fluorescence spectra are treated as two data collection methods that are added to queue for execution.

In *MXCuBE2*, the measurement of absorption-edge (XANES; X-ray absorption near-edge structure) scans and X-ray fluorescence (XRF) spectra (Leonard *et al.*, 2009 ▸) are treated as any other data collection (*i.e.* they are added to the queue for subsequent execution) (Fig. 7 ▸). Absorption-edge scans can be set up by choosing the relevant element from a periodic table and, if applicable, defining which of the element’s *L*-edges within the reach of the beamline energy range should be targeted. The scan itself is then performed with the lowest photon flux that produces a fluorescence signal above a predefined threshold as measured on the beamline-specific fluorescence detector. The results of the scan are displayed in the corresponding ‘Details’ sub-tab, and the energies for the peak and inflection point obtained after data fitting by *CHOOCH* (Evans & Pettifer, 2001 ▸) are extracted for potential use in ensuing data collections applied to the selected sample. The energy for any ‘remote’ data set is taken as 50 eV above the energy of the peak. It is thus possible, for example, to construct a data collection queue comprising the calculation of a data collection strategy using characterization, the measurement and processing of an absorption edge scan and, finally, standard data collections using the photon energies (peak, inflection point and remote) derived from this. Peak, inflection point and remote energies are automatically stored for the selected sample after each energy scan.

X-ray fluorescence spectra are recorded and displayed in a similar way and automatically fitted using the *PyMCA* package (Solé *et al.*, 2007 ▸). As for any other data collection, results are displayed in the ‘Details’ window. Here, moving of the cursor highlights the energy of emission lines and shows the element most likely to have produced it. Absorption-edge scans, X-ray fluorescence spectra and their results are also stored and displayed in the *ISPyB* database.

#### 4.3.5. Advanced data collection   

The data collection methods included in this area represent more complex data collection protocols that may require input from external data analyses and/or ‘experiment descriptors’ to define the actions to be carried out in subsequent steps of the protocol selected. This can be performed using different decision-making tools. The access to these methods is achieved via the XML-RPC server (§3.3). Installation of the optional methods available in this tab diverges at the different sites where *MXCuBE2* is installed, matching local needs and the different configuration of the beamlines.

### 4.4. Site-specific installations and partner contributions   

#### 4.4.1. ESRF   

The ESRF Structural Biology Group MX beamlines [ID23-1 (Nurizzo *et al.*, 2006 ▸), ID23-2 (Flot *et al.*, 2010 ▸), ID29 (de Sanctis *et al.*, 2012 ▸), ID30A-1 (MASSIF-1) (Bowler *et al.*, 2015 ▸), ID30A-3 (MASSIF-3) (Mueller-Dieckmann *et al.*, 2015 ▸), ID30B (McCarthy *et al.*, 2018 ▸)] run *MXCuBE2* in the Qt3 framework interfaced with the Passerelle workflow engine (https://supportsquare.io/) which manages complex experiments and runs on the Beamline Expert System (BES) server. The BES is a customized version of the Passerelle Enterprise Decision Manager (EDM) that runs the requested workflow on a separate computing cluster. The workflow communicates with *MXCuBE2* via the XML-RPC protocol (§3.3) and requests data collections or motor movements (for example, goniometer motors). The different steps of a workflow execution are consecutively executed after either a decision point following the analysis of the data recorded in the previous step or a request for user intervention. Thanks to the ease of the design and the versatility of the XML-RPC communication protocol, the available workflows at the ESRF’s MX beamlines are constantly evolving in order to include novel methods and accommodate requests from the user community. The most popular workflows include mesh scans and X-ray centring methods, which are mainly used in different flavours of hands-off data collection protocols (Svensson *et al.*, 2015 ▸) and in micro-crystallography experiments such as Mesh&Collect (Zander *et al.*, 2015 ▸). Dedicated workflows also facilitate use of multi-axis goniometers, particularly in the calculation of the MK3 mini-κ goniometer angles required to adjust the diffraction geometry needed to optimize the total rotation angle to minimize spot overlap or to record Friedel pairs on the same diffraction image (de Sanctis *et al.*, 2016 ▸; Brockhauser *et al.*, 2013 ▸). Workflows are also very important in the design and execution of elaborate data collection strategies (*i.e.* inverse beam, interleaved MAD/SAD) for experimental phasing protocols exploiting anomalous dispersion. *MXCuBE2* is also used to navigate and collect diffraction data from *in situ* crystals using a crystallization plate holder (Arinax, France) on ID30B (McCarthy *et al.*, 2018 ▸). Together with the MAX IV team (§4.4.4), the ESRF has developed the latest version of the user interface *MXCuBE3* (Mueller *et al.*, 2017 ▸), which has already been proposed to the users of ID29 and ID23-2 and can be used for remote-access experiments by using a recent web browser. The installation on the other ESRF beamlines was completed by the end of 2018.

#### 4.4.2. SOLEIL – PROXIMA-1 and PROXIMA-2A   

In line with the implementation of *MXCuBE2* at the ESRF, PROXIMA-1 (PX1) and PROXIMA-2 A (PX2-A) at SOLEIL operate the GUI in the Qt3 framework. Although highly similar overall, slight differences exist at the versions of *MXCuBE2* installed at the two beamlines due to a difference in beamline hardware and a variability in the scientific specializations. At PX1 and PX2-A, the overall integrity of the diffractometer motors is independently controlled via TANGO devices (Chaize *et al.*, 1999 ▸; http://www.tango-controls.org/), which are, for the majority, linked to and displayed within *MXCuBE2*. One major hardware difference on PX1 with other beamlines lies in the specific implementation of a three-axis goniometer based on a κ-geometry. To properly operate the diffractometer while keeping the entire hardware safe from collisions, protocols have been developed to rapidly control the experiments while considering a sphere of collision and safety location of all instruments around the sample position. Consequently, users are provided with only accessible positions for each component based on the positions and input of other diffractometer components, including the beam-stop position, the presence or absence of a pre-sample capillary, the κ-angle of the goniometer, the targeted energy for the data collection and the targeted resolution (both of which have an influence on the sample-to-detector and sample-to-beam-stop distances).

#### 4.4.3. HZB (BESSY II)   

At the BESSY II electron storage ring, two energy-tuneable MX beamlines and one fixed-energy station (Mueller *et al.*, 2015 ▸, 2012 ▸) are currently operated by the HZB-MX group. Both energy tuneable beamlines, BL14.1 and BL14.2, are controlled by *MXCuBE2* featuring the Qt4-based user interface. A further installation of *MXCuBE2* is anticipated in the scope of the experimental-station upgrade of the fixed-energy beamline BL14.3 scheduled for early 2019. Although the end-station hardware (including the main components such as the diffractometer and the sample-transfer robot) differ significantly between beamlines, an almost identical user interface can be offered to the beamline users thanks to the *MXCuBE2* hardware abstraction layer. Beamline BL14.1 features a MD2-microdiffractometer (Arinax, France), a CATS sample changer (IRELEC, France) and a PILATUS 6M detector (DECTRIS, Switzerland). In order to integrate the CATS sample changer, a new hardware object had to be developed. Recently, a plate manipulator (Arinax, France) has been installed, which will soon be integrated and will allow *in situ* crystal screening and data collection. In order to support the GROB sample-changing robot (NatX-ray, France) as well as the piezo-controlled beam-shaping devices and the fast air-bearing axis of the Nanodiff-diffractometer on HZB’s most recent MX beamline BL14.2, new hardware object software modules were added to the pool of supported control devices. *MXCuBE2* at HZB (BESSY II) currently supports crystal characterization by *EDNA* and standard data collection workflows with full sample-changer integration well suited for routine data acquisition, *e.g.* in fragment-screening campaigns (Huschmann *et al.*, 2016 ▸). These workflows are complemented with XANES scans and energy-dispersive spectra using an Amptek silicon-drift fluorescence detector for *de novo* structure solution applications.

In order to enhance the locally available experimental options for users, the integration of the helical data collection protocol as well as the two-dimensional grid scans has started and the release of this for standard user experiments is expected in early 2019. It is also planned to link the *XDS* data processing expert system *XDSAPP* (Sparta *et al.*, 2016 ▸), developed at HZB (BESSY II) directly to data acquisition via *MXCuBE2*.

#### 4.4.4. MAX IV   

At the old MAX-lab (MAX II), *MXCuBE2* was used to control the experimental setup of the former energy-tuneable MX-beamline 911-3 until its decommissioning at the end of 2015. This included the control of the main beamline functions and support of the experimental station environment including: an MD2 micro-diffractometer, a CATS sample changer (IRELEC, France) and a Rayonix 225 CCD detector (Rayonix, USA). As a result of the successful *MXCuBE* collaboration, full support of the CATS sample changer using an ALBA developed TANGO device server and a HZB developed CATS hardware object was implemented and utilized. Furthermore, a cryo-shutter designed by HZB was integrated into *MXCuBE2* for crystal annealing. With the setup of remote access in *MXCuBE2*, the MAX IV team together with SOLEIL could perform different data collection experiments remotely. Data characterization by *EDNA* and data evaluation using the *GrenADeS* [developed by ESRF (Monaco *et al.*, 2013 ▸)] and *EDNA* processing pipelines (Incardona *et al.*, 2009 ▸) were used during user operations on a daily basis. The MAX IV team developed their own hardware object and evaluation environment for carrying out XRF scans using an Amptek silicon drift detector. Finally, all samples and related data acquisition and processing results were stored in the *ISPyB* LIMS database, and were accessible to users after their experiments.

At the new MAX IV synchrotron (3 GeV storage ring), the MX team has been developing the next generation interface, *MXCuBE3*, in close collaboration with the ESRF (Mueller *et al.*, 2017 ▸). The new *MXCuBE3* has been deployed at the first MX-beamline (BioMAX) of the MAX IV Laboratory (Thunnissen *et al.*, 2013 ▸) and is used as the beamline control GUI during user experiments. While the hardware object layer of *MXCuBE3* has been inherited from *MXCuBE2*, preserving a high downstream compatibility, a completely new and user-friendly interface for *MXCuBE3* has been conceived as a web application. Within its current implementation, *MXCuBE3* supports the MD3 micro-diffractometer (Arinax, France) and the EIGER 16M hybrid pixel detector (DECTRIS, Switzerland), providing standard data collections, including helical scans and the basic beamline functions. Control of the ISARA (IRELEC, France) sample changer and additional features, including raster scans, XRF scans, XANES, workflows and remote access are currently being developed and will be implemented in the near future.

#### 4.4.5. EMBL-Hamburg   

Two MX beamlines, P13 and P14, run by the European Molecular Biology Laboratory (EMBL) at the PETRA III storage ring are currently using *MXCuBE2* featuring a graphics layer built with Qt4 libraries. The migration to Qt4 of the core Qt-components of the *BlissFramework* (such as configuration tools, GUI designer and GUI supervisor) was contributed to the collaboration by EMBL-Hamburg. The core endstation components are an MD2 diffractometer (Arinax, France) and a PILATUS 6M-F detector (DECTRIS, Switzerland) at P13, and an MD3 diffractometer (Arinax, France) and an EIGER 16M detector (DECTRIS, Switzerland) at P14. Both beamlines operate cryogenic ‘MARVIN’ sample changers developed at EMBL-Hamburg (Fiedler *et al.*, unpublished). On both diffractometers, mini-κ goniostats (Brockhauser *et al.*, 2013 ▸) are installed and supported by *MXCuBE2*. At P14, *MXCuBE2* provides additional support to the operation of the CrystalDirect^TM^ (Cipriani *et al.*, 2012 ▸) plates with the plate manipulator when this is mounted on the MD3.

In contrast to other sites involved in the *MXCuBE* Collaboration, at P13 and P14 *MXCuBE2* communicates with beamline devices via the *TINE* (Bartkiewicz & Duval, 2007 ▸) control system. Furthermore, data acquisition sequences at the ‘Start/Execute’ step are delegated to middleware *TINE* services which tightly coordinate the actions of a multitude of devices, as briefly described in Karpics *et al.* (2016 ▸). For the ‘Collection methods’, *MXCuBE2* at P13/P14 implements standard, helical and mesh data collections, sample characterization, and XRF and energy scans. In these methods, automated data analysis is triggered and the results are deposited in *ISPyB*. For rotation data processing, *EDNAproc* (Monaco *et al.*, 2013 ▸), *autoPROC* (Vonrhein *et al.*, 2011 ▸) and *XIA2* (Winter, 2010 ▸) are used.

When executing queues consisting of standard or helical collections, *MXCuBE2* provides an option for interleaved – as opposed to sequential – execution of queue elements. Thus, interleaved collections at different energies, rotation angles, κ-diffractometer settings, sample centrings, or any other collection setting are achieved via a straightforward and transparent interface.

Under the ‘Advanced’ collection method, mesh and serial helical-scan data collections [mesh scans with larger rotation angles per line (Gati *et al.*, 2014 ▸)] are offered. With small and intense beams, in combination with a high-frame-rate detector at P14, such collections often involve many tens of thousands of frames, requiring the customization of the representation of meshes. After delegating the scan execution to middleware *TINE* services (and further to the low-level hardware control), *MXCuBE2* triggers automatic diffraction detection using the program *DOZOR* (Popov & Bourenkov, 2016 ▸) encapsulated in the *EDNA* plugin (Incardona *et al.*, 2009 ▸). *MXCuBE2* receives the results of the *DOZOR* analysis via XML-RPC and constructs and presents a diffraction heat map on-the-fly. The heat map is linked to the diffraction image display and provides a broad range of interactive functionalities for analysis and definition of centring points for subsequent data collections.

At the EMBL-Hamburg beamlines, *MXCuBE2* implements a number of customized features for automatic re-configuration of the beamline optics and for automatic beam centring. For instance, when P14 operates in a collimated regime, the shape of the (top-hat) beam at the sample is defined by dragging a rectangle over the sample on the video display.

Remote access to the complete data collection environment at P13/P14, including the single instance of *MXCuBE2* running at each beamline, is provided via the *TeamViewer* (https://www.teamviewer.com) remote desktop software.

#### 4.4.6. ALBA   

At ALBA, *MXCuBE2* is the user interface for the MX beamlines, XALOC (Juanhuix *et al.*, 2014 ▸) and XAIRA at the ALBA synchrotron. *MXCuBE2* has been recently implemented for regular use at the operational beamline XALOC after an extended commissioning phase with external users. XAIRA, which is the microfocus beamline currently under construction at ALBA, will also benefit from the implementation of *MXCuBE2*, even though the hardware for this beamline differs substantially from that installed at XALOC.

The current implementation of *MXCuBE2* at XALOC is built on top of the Sardana/Taurus control system and TANGO (Reszela *et al.*, 2014 ▸, 2017 ▸; Fernández-Carreiras *et al.*, 2011 ▸; Pascual-Izarra *et al.*, 2015 ▸). The hardware installed on the beamline is accessed through the Sardana layer or the TANGO Device Server (DS) and linked to the *MXCuBE2* user interface. Different generic and specific hardware objects for the current hardware are used as connectors between the native control system and the *MXCuBE2*/Qt4 interface, while standard collection methods are implemented using the *Sardana Scan Framework*. The overall status of the beamline is managed with a specific Beamline Supervisor TANGO DS. This supervisor defines a set of beamline configurations (or phases) corresponding to different operation procedures such as ‘sample change’, ‘diffraction’ or ‘beam visualization’, providing a robust machine state and safe operation. Furthermore, this implementation allows for a smooth future transition from *MXCuBE2* to the web-based *MXCuBE3*.

Test images are processed using *EDNA* running on a computing cluster at ALBA, and the results are uploaded to the *ISPyB* database and made available to users. Collected data are processed on the cluster using the *autoPROC* program from Global Phasing Ltd (Cambridge, UK) (Von­rhein *et al.*, 2011 ▸). The default data processing is performed using standard parameters, with default resolution cutoffs based on CC1/2. The user can request a change of any of the processing parameters. The data can conveniently be reprocessed and phased using molecular replacement [*Phaser*, (McCoy *et al.*, 2007 ▸)] or *ARCIMBOLDO* (Rodríguez *et al.*, 2009 ▸) using *Xamurai* (unpublished), an in-house-developed program.

In the near future, the implementation of *MXCuBE2* at XALOC will be expanded to include automated beam-conditioning options such as the adjustment of slits and apertures, and to offer more complex and tailored methods of data collection, including interleaved data collections. XAIRA will implement these developments while also including options specific to its scientific case and final hardware configuration.

#### 4.4.7. Global Phasing Ltd   

Global Phasing Ltd (GPhL) is the only non-synchrotron member of the *MXCuBE* Collaboration. Its involvement in joint developments with synchrotrons dates back to its participation in the BIOXHIT project, with a special interest in the data processing aspects of the deployment of multi-axis goniometry for experimental phasing that was taking place at that time. Since then, as part of its activities in developing improved and better-integrated software for using MX as a tool for structure-based drug discovery, with support from the pharmaceutical industry for the past 20+ years, GPhL has pursued sustained efforts towards finding ways of increasing the quality of X-ray data collected for high-throughput ligand screening by crystallography. High degrees of automation have been achieved towards this goal, *e.g.* on the MASSIF-1 (ESRF ID30A-1) (Bowler *et al.*, 2015 ▸) and XChem (Diamond I04-1) beamlines, resulting in protocols that are very time-efficient by being ‘minimalistic’. This entails skipping steps such as the design of strategies using multiple orientations adapted to the symmetry and orientation of each individual crystal. The rationale for this choice is that although such steps are known to have the potential of significantly boosting the quality of the final datasets, they would, in the current state of affairs, require human intervention and thus disrupt automation. GPhL’s work on the implementation of fully automated expert protocols was initiated in July 2013 as part of a collaboration with Diamond focused on the I23 beamline, on which the curved geometry and the resulting aspect ratio of the PILATUS 12M detector made it absolutely necessary to use multiple-orientation strategies and to make their design completely automatic, with built-in avoidance of collisions as well as anticipation and remediation of shadowing by the goniometer. For reasons both methodological (viz. aiming at a maximally generic and transferable implementation) and practical (the GPhL funding model, emphasizing the need for maximum ease of deployment on a variety of beamlines at multiple synchrotrons) it was decided that the software should take the form of a unique workflow, embodying all the logic and expertise needed to design optimal multi-orientation strategies on-the-fly and to supervise their execution by directly communicating with the beamline control software (BCS), including, crucially, the automatic re-centring of the sample after each change of orientation. A major milestone of this collaboration was reached in September 2016, when the GPhL workflow steered a completely automated three-wavelength interleaved MAD experiment on a lysozyme crystal (including the alignment of its fourfold axis and the use of a cusp-filling orientation) on the Diamond I04-1 beamline. This steering took place through a message bus connecting the workflow with GDA (the Diamond BCS), based on an abstract beamline interface intended to make the task of writing similar connections with other BCSs as simple as possible.

Work aimed at creating such a connection with *MXCuBE2* began in the summer of 2017 and was able to progress rapidly thanks to access to the *MXCuBE2* source code and to its developers, provided by membership of the *MXCuBE* collaboration. In particular, the need to enlarge the internal execution queue of the old *MXCuBE* to accommodate the ‘third-party design and control’ paradigm proposed by GPhL was recognized and assigned a high priority by the developers, and that enlargement was a major structural component of the transition to *MXCuBE2*. The first live tests of this connection (with the Qt3 version of *MXCuBE2*) took place on the ESRF ID30B beamline in October 2017, and by May 2018 the GPhL team was able to successfully run remotely a similar (fully automated three-wavelength interleaved MAD) experiment to the one previously performed at Diamond. Work is underway to extend the scope of this connection so as to include the Qt4 version of *MXCuBE2* (on beamlines at ALBA and SOLEIL) and to *MXCuBE3*.

### 4.5. Remote access   

Both *MXCuBE* and *MXCuBE2* offer integrated remote access features (Gabadinho *et al.*, 2010 ▸) to the beamlines where they are installed. In the current remote access set-up, access to the local control computer is performed via dedicated software such as *NX NoMachine*, which connects to a dedicated machine inside the synchrotron site firewall and from there to the local control machine. Although remote control of MX beamlines is increasing, performance can suffer from deterioration in connection speed. In order to facilitate and improve remote access, *MXCuBE* is currently being redesigned as a web application called *MXCuBE3*.

*MXCuBE3* simplifies and speeds up remote-access procedures as it will not require any installation on the client (remote) side other than a recent version of a web browser and the ability of the client to connect to the beamline via a predefined port. Currently available at the BioMAX beamline of the MAX IV Laboratory (§4.4.4) and at the ID29 and ID23-2 beamlines at the ESRF (§4.4.1), *MXCuBE3* is platform independent and uses the same hardware repository as *MXCuBE2*, making it possible, without any change in configuration, to run either *MXCuBE2* or the new web-based application on the same control computer. While a preliminary report on the design of *MXCuBE3* has already been published (Mueller *et al.*, 2017 ▸), further details of this new avenue for the *MXCuBE* Collaboration will be given in due course.

## 5. Discussion   

The *MXCuBE* Collaboration brings together different partners aiming for the development of a common beamline control environment for macromolecular crystallography experiments. The European MX user-community takes advantage of finding a very similar and intuitive graphical interface when performing their experiments at different beamlines. Currently the collaboration is developing a new interface (*MXCuBE3*) that can ensure the future sustainability of *MXCuBE* and permit the evolution of the graphical user interface over time. The software architecture is explicitly designed to facilitate the deployment on any hardware controller layer at different synchrotron sites and the necessary development for the implementation of new hardware and new data collection methods can easily be adopted by all *MXCuBE* Collaboration partner sites. This agility allows *MXCuBE* to be ready for the future challenges in MX, and of serial crystallography at synchrotrons in particular.

Starting from the early implementations at X-ray free-electron laser sources (Chapman *et al.*, 2011 ▸), MX is experiencing a paradigm shift in approaches to data collection, where the traditional dogma of ‘one crystal, one data collection’ is being replaced by more complex, yet more efficient, strategies using multiple crystal orientations of a single crystal (Weinert *et al.*, 2015 ▸; Klinke *et al.*, 2015 ▸), multiple crystals to increase the multiplicity (and hence statistical reliability) in anomalous data collection (Liu *et al.*, 2012 ▸), right through to serial crystallography methods (Standfuss & Spence, 2017 ▸). These serial methods, at least for the present, can be viewed as complementary to (and not a replacement for) more traditional data collection, offering methods of extracting useful information from crystals of micrometre dimensions or smaller, being able to mitigate the effects of radiation damage by sharing dose over many crystals (which exhibit presumably identical internal molecular structures – the ultimate case of isomorphism), and even permitting the extraction of usable anomalous signal for phasing (Zander *et al.*, 2015 ▸; Schlichting, 2017 ▸). Although the extent of the role of such methods in the crystallographers’ armoury is as yet unclear, it is certain that the ‘book-keeping’ of highly multiple-crystal data collection represents a major challenge. For example, the automatic presentation of many expected crystal positions (from a grid or plate) along with the registration of the necessary parameters (rotation angle, oscillation range if applicable and their link to an automatically assigned image number *etc*.) for many hundreds or indeed thousands of positions will be essential in order to pave the way for future automatic data collections and subsequent processing. *MXCuBE* and the different software programs that work in association with it (*ISPyB*, different automatic data analysis pipelines such as *XIA2*, *autoPROC*, *XDSME*, *XDSAPP*, *GrenADeS* *etc*.) will require major changes based on a generic data model which includes the possibility of interleaving multiple data collections (energy, orientation) with multiple samples (from several up to many thousands) coupled with rapidity and fluidity of a user interface and the correct level of information about the progress of the different types of data collection, permitting decision making by the user.
